# The Effects of Oral Anticoagulant Exposure on the Surgical Outcomes of Patients Undergoing Surgery for High-Risk Abdominal Emergencies

**DOI:** 10.1007/s11605-021-04964-9

**Published:** 2021-03-22

**Authors:** Woubet Tefera Kassahun, Tristan Cedric Wagner, Jonas Babel, Matthias Mehdorn

**Affiliations:** Faculty of Medicine, Clinic for Visceral, Transplantation, Thoracic and Vascular Surgery, University of Leipzig, Liebig Strasse 20, 04103 Leipzig, Germany

**Keywords:** High-risk emergency, Surgery, Anticoagulants, Bleeding, Thromboembolism

## Abstract

**Background:**

In chronic anticoagulant users undergoing surgery, bleeding and thromboembolism are common and serious complications. Many studies on mainly elective or minor emergency surgical procedures with low associated risks have focused on these outcomes. In comparison, patients undergoing high-risk emergency abdominal surgical procedures have not received sufficient attention. This study aimed to compare outcomes between oral anticoagulant users and nonusers who required emergency laparotomy for high-risk abdominal emergencies.

**Methods:**

Patients who underwent surgery for abdominal emergencies at our institution between January 2012 and July 2019 were retrospectively reviewed.

**Results:**

There were 875 patients, including 370 anticoagulant users and 505 nonusers. Of the 370 anticoagulant users, 189 (51.3), 77 (20.8%), 45 (12.2%), and 59 (15.9%) were prescribed antiplatelets, a vitamin k antagonist, a direct oral anticoagulant, and a combination drug regimen, respectively. The most common high-risk emergencies requiring surgery in both groups were perforated viscus (25.7% vs 40.9%), mesenteric ischemia with enteric necrosis (27% vs 12.8%), and bowel obstruction (17.6% vs 28.1%). The overall bleeding rate was higher (29.2% vs 22%, p = 0.015) in anticoagulant users than in nonusers, but the major bleeding rate was similar (17.8% vs 14.1%, p = 0.129) between the two groups. The rates of thromboembolic events and mortality were significantly higher in anticoagulant users than in nonusers (25.7% vs 9.7%, p < 0.0001 and 39.7% vs 31.1%, p = 0.01, respectively). Liver cirrhosis, peripheral arterial diseases, reoperation, and blood product transfusion were independent predictors of the overall risk of bleeding or TEEs, according to the multivariate analysis. In this model, liver cirrhosis had the largest overall effect on mortality, followed by pneumonia, thromboembolism, peripheral arterial disease, blood product transfusion, and atrial fibrillation. The use of oral anticoagulants was not an independent predictor of either bleeding or in-hospital mortality. The use of oral anticoagulants was associated with a decreased risk of all-cause in-hospital mortality.

**Conclusion:**

Based on our results, the continued use of oral anticoagulants is more protective than harmful considering the overall outcomes in this subset of patients.

**Supplementary Information:**

The online version contains supplementary material available at 10.1007/s11605-021-04964-9.

## Introduction

Oral anticoagulant medication is indicated as preventive therapy for thromboembolic disease in patients with cardiovascular comorbidities 1. This includes patients with an increased risk of coronary artery disease, patients treated with percutaneous coronary stents or mechanical prosthetic heart valves, patients with a history of venous thromboembolism, and the majority of patients with chronic atrial fibrillation (AF) to prevent stroke.

In patients exposed to oral anticoagulants (OACs) who require emergency surgery, the risks for bleeding, thromboembolism, and mortality are multifold higher than those in patients undergoing elective surgery 2.

Anticoagulants can be discontinued in preparation for an elective procedure. However, it is impossible to implement such a practice when patients require emergency surgery.

For the elective surgical spectrum, the risks of bleeding, thromboembolism, and mortality have been assessed in anticoagulated patients who require surgery 2–6.

Studies examining the outcomes of patients exposed to OACs who required urgent surgery 2, 7–9 included patients with all types of emergencies (orthopedic, vascular, thoracic, urologic, abdominal) and subsets (up to 40%) of patients with diagnostic and minor same-day procedures.

As a result, there is a paucity of information on the outcomes of anticoagulated patients who require surgery for high-risk abdominal emergencies.

The aim of the present study is therefore to compare outcomes focusing on bleeding, thromboembolic events (TEEs) and all-cause in-hospital mortality between anticoagulant users and nonusers who required emergency laparotomy (EL) for high-risk abdominal emergencies.

## Methods

The patients included in this study were derived mainly from our previous study, in which we analyzed the effects of reoperation on surgical outcomes 10; however, in this study, we focused on patients exposed to OACs who required surgery for high-risk abdominal emergencies.

A retrospective review of patients who underwent surgery for abdominal emergencies at our institution was undertaken. The retrospective review timeframe was from January 2012 to July 2019. In this period, 2484 patients underwent 3563 emergency abdominal operations, which were defined as any surgery that needed to be performed as soon as possible during the same admission that the diagnosis was made.

High-risk abdominal emergencies were defined as those that had an anticipated high risk of in-hospital death due to septic and/or hemorrhagic complications and required emergency surgery. As had been addressed previously in other studies as well 11–13, we set the threshold for high risk as a predicted mortality rate of greater than or equal to 10%.

**Fig. 1:**
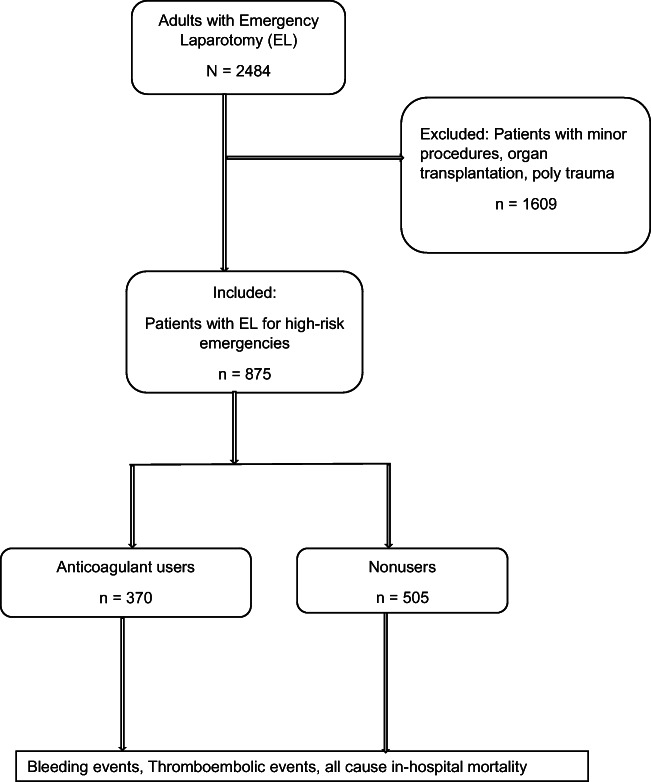
Flow chart of study cohort

The patient selection criteria are depicted in Fig. 1.

After applying the exclusion criteria, the remaining 875 patients were divided into two groups based on whether they had chronic exposure to OACs and antiplatelets. Patients taking OACs and antiplatelets at the time of their latest office visit or hospital admission just prior to the emergency operation (N = 370) were the focus of this study. For the purposes of this study, surgical patients taking any of the following baseline anticoagulants and antiplatelets alone or in combination for various indications were considered anticoagulant users: (1) antiplatelets (acetylsalicylic acid, clopidogrel), (2) a vitamin k antagonist ([VKA] phenprocoumon), (3) direct oral anticoagulants (DOACs [apixaban, rivaroxaban, edoxaban, or dabigatran]), or (4) a combination drug regimen (acetylsalicylic acid with any of the above). We divided anticoagulants in this way because there were too few patients to subdivide by a specific type of drug. We determined the drug use status by reviewing the medication record at the time of hospital admission and the operation report from the surgeon.

Patients who underwent emergency surgery and were not taking anticoagulant medication (N = 505) comprised the reference group. The patient demographics, perioperative details, and clinicopathologic factors were collected retrospectively from patient charts.

We categorized the index procedures into 9 categories and decided to forego statistical analysis because the sample size of each prespecified abdominal surgical procedure was too small for meaningful statistical results.

Depending on the patient’s renal function, when oral anticoagulant treatment was interrupted, bridging therapy was initiated with unfractionated or low molecular weight heparin (UFH, LMWH) after a case-by-case risk assessment until the OAC could be readministered. The timing of anticoagulant readministration was at the discretion of the treating physicians.

Postoperative complications were recorded and then graded using the Clavien-Dindo (CD) classification 14. Based on this classification, the comprehensive complication index (CCI) was calculated to best mirror the burden of each complication.

The major study outcomes were as follows: (1) the occurrence of bleeding events (major and minor), (2) the occurrence of TEEs, and (3) all-cause in-hospital mortality.

Major bleeding was defined as bleeding that was clinically apparent, resulting in a hemoglobin decrease of > 2 g/dL or requiring a transfusion of more than 2 units of packed red blood cells 15.

TEEs included deep venous thrombosis, pulmonary embolism, myocardial infarction, ischemic stroke, and systemic embolism. We included all bleeding and thromboembolic events that occurred at any time (pre-, intra-, and/or postoperative) during hospitalization.

We evaluated the use of blood products (packed red blood cells, pooled platelets, fresh frozen plasma) and prothrombin complex concentrates (PCCs) during the initial admission, during the surgical procedure or after surgery until discharge using electronic chart records.

Summary statistics were obtained using established methods as described in detail previously 10. Categorical data are summarized as percentages, and differences between groups were tested with the chi-square test. Continuous variables are summarized as the means and standard deviations or medians and interquartile ranges, and differences between groups were tested using Student’s *t*-test. Multivariate analysis was performed using logistic regression. The odds ratios (ORs) and 95% confidence intervals (CIs) were estimated. Statistical significance was defined by a 2-sided p ≤ 0.05. Statistical analysis was performed using SPSS version 25 software (IBM Corporation, USA).

Ethical approval from the local institutional review board was obtained for this study.

## Results

The baseline characteristics of the patients and comorbidities are summarized in Table 1.

**Table 1:** Baseline patient characteristics

Group	Anticoagulant users (*N* = 370)	Nonusers (*N*= 505)	*p* value
Age, years, mean ± SD	72.52 ± 11.93	59.90 ± 17.91	< 0.0001
BMI, mean ± SD	27.38 ± 5.85	26.05± 7.09	0.003
Men (%)	217 (58.6)	280 (55.1)	0.316
COD, mean ± SD	7.47 ± 2.61	3.56 ± 2.08	< 0.0001
ASA ≥ 3	316 (90)	339 (69.9)	< 0.0001
Major comorbidities			
Hypertension	328 (88.6)	251 (49.7)	< 0.0001
Diabetes	124 (33.5)	92 (18.2)	< 0.0001
Coronary artery disease	139 (37.6)	20 (4.0)	< 0.0001
Congestive heart failure	124 (33.5)	52 (10.3)	< 0.0001
Atrial fibrillation	199 (53.8)	66 (13.1)	< 0.0001
Peripheral artery disease	162 (43.8)	58 (11.5)	< 0.0001
COPD	76 (20.5)	53 (10.5)	< 0.0001
Chronic renal failure	110 (29.7)	43 (8.5)	< 0.0001
Liver cirrhosis	24 (6.5)	52 (10.3)	0.048
CND	119 (32.2)	86 (16.9)	< 0.0001
Malignancy	94 (25.4)	136 (26.9)	0.613
Anticoagulants			
Acetylsalicylic acid	179 (48.4)	―	―
Clopidogrel	10 (2.7)	―	―
VKA	77 (20.8)	―	―
DOAC	45 (12.2)	―	―
Combination^¥^	59 (16.0)	―	―

*N* total number of patients, *SD* standard deviation, *COD* coexisting disease per patient, *COPD* chronic obstructive lung disease; CND indicates central nervous system disease and holds for patients with medically documented cerebral vascular accident, transient ischemic attack, or neurological deficit of central origin; *VKA* vitamin K antagonists, *DOAC* direct oral anticoagulants. Numbers in bracket show values presented in *n* (%) unless noted otherwise

^¥^Indicates a combination of acetylsalicylic acid with any of the indicated oral anticoagulants

Overall, the patients who underwent unplanned EL were comparable between groups in terms of sex, but the anticoagulant users were significantly older, had a significantly higher comorbidity burden from all of the comorbidities evaluated except liver cirrhosis, and had a higher body mass index (BMI) and American Society of Anesthesiologists (ASA) class than the nonusers.

Of the 370 anticoagulant users, 191 (51.1) were prescribed antiplatelets (acetylsalicylic acid, n =179 [48.4]; clopidogrel, n = 10 [2.7]), 77 (20.8%) were prescribed a VKA, 45 (12.2%) were prescribed a DOAC, and 59 (16%) were prescribed a combination drug regimen (acetylsalicylic acid + clopidogrel, n = 41; ASA + a DOAC, n = 9; ASA + a VKA, n = 9).

The primary operative indications and initial surgical procedures are displayed in Table 2. Additionally, the procedure-specific outcome characteristics of the patients are shown in a supplemental table.

**Table 2:** Primary Indications for surgery and initial surgical procedures by group

	Anticoagulant users	Nonusers
	*N* = 370	*N* = 505
Surgical emergency		
Perforated viscus	95 (25.7)	208 (40.9)
Mesenteric ischemia	100 (27.0)	65 (12.8)
Bowel obstruction	65 (17.6)	143 (28.1)
Hemorrhage	32 (8.6)	42 (8.3)
Complicated cholecystitis	38 (10.3)	12 (2.4)
Complicated appendicitis	22 (5.9)	7 (1.9)
Complicated pancreatitis	4 (1.4)	10 (2.0)
Toxic mega colo	7 (1.9)	7 (1.4)
IAS	7 (2.0)	8 (1.8)
Abdominal compartment	--------	6 (1.2)
Initial access to abdomen		
Minimally invasive	96 (25.9)	134 (26.4)
Open	274 (74.1)	374 (73.6)
Conversion rate*	42 (43.6)	61 (45.5)
Index procedure		
Bowel resection with primary anastomosis	70 (19)	76 (15.1)
Bowel resection without primary anastomosis	78 (21.1)	100 (19.8)
Closure of viscus organ	36 (9.7)	94 (18.6)
Surgery for complicated appendicitis	23 (6.2)	6 (1.2)
Surgery for complicated cholecystitis	38 (10.3)	12 (2.4)
Extensive adhesiolysis for bowel obstruction	29 (7.8)	75 (14.8)
Control of hemorrhage	22 (5.9)	23 (4.5)
Multiple procedures	40 (10.8)	72 (14.4)
Miscellaneous procedures	34 (9.2)	47 (9.3)

*IAS* intraabdominal sepsis with multiple abscesses. Numbers in bracket indicate values presented in *n* (%)

*Indicates conversion of minimally invasive start to open procedure

The most common indications in both groups included perforated viscus, mesenteric ischemia, and bowel obstruction. The most common surgical procedure was bowel resection with or without primary anastomosis in both groups.

Additionally, there were no significant differences between anticoagulant users and nonusers regarding the initial surgical approach: minimally invasive start (25.9% vs 26%), open procedure (74.1% vs 73.6%), and conversion from a minimally invasive start to an open procedure (43.6% vs 45.5%). A comparison of perioperative events and surgical outcomes between anticoagulant users and nonusers is summarized in Table 3.

**Table 3:** Summary of outcomes

Group	Anticoagulant users (*N* = 370)	Nonusers (*N*= 505)	*p* value
Complications	280 (75.7)	351 (69.6)	0.049
COMP, mean ± SD	5.58 ± 3.49	5.03 ± 3.53	0.047
CCI, mean ± SD	61.33 ± 36.37	52.53 ± 37.22	0.001
Overall bleeding events	108 (29.2)	111 (22.0)	0.015
Major bleeding	66 (17.8)	71 (14.1)	0.129
BPT	114 (30.8)	111 (22.0)	0.003
PRBC, units, mean ± SD	6.24 ± 5.68	6.53 ± 6.99	0.744
PL, units, mean ± SD	2.23 ± 2.06	3.18 ± 3.0	0.141
FFP, units, mean ± SD	7.66 ± 6.27	8.38 ± 7.10	0.556
PCC, ITU, mean ± SD	2426 ± 1386	2317 ± 1520	0.838
Thromboembolic events	95 (25.7)	49 (9.7)	< 0.0001
Pulmonary embolism	8 (2.2)	16 (3.2)	0.371
CVI	12 (3.2)	12 (2.4)	0.444
Myocardial infarction	14 (3.8)	3 (0.6)	0.001
Acute renal failure	143 (38.6)	150 (30.0)	0.008
Liver failure	90 (24.3)	112 (22.2)	0.429
ICU	307 (83.0)	361 (71.5)	< 0.0001
ICU-LOS, days, median (IR)	6 (1-127)	4 (1-135)	
MV	227 (61.4)	262 (51.9)	0.005
DMV, hours, median (IR)	61 (1-1713)	59 (1-865)	
Relaparotomy	144 (38.9)	170 (33.7)	0.109
In-hospital death	147 (39.7)	157 (31.1)	0.010
LOS, days, median (IR)	13 (1-155)	13 (1-200)	

*COMP* complications per patient, *BPT* blood product transfusion, *PRBC* packed red blood cells in units, *PL* pooled platelets in units, *FFP* fresh frozen plasma in units, *PCCs* prothrombin complex concentrates, *IU* international unit, *CVI* cerebrovascular ischemic event, *CCI* comprehensive complication index, *ICU* intensive care unit, *MV* mechanical ventilation, *DMV* duration of mechanical ventilation, *IR* interquartile range, *LOS* length of hospital stay defined as the time from the date of the initial admission to the date of discharge, transfer to external services, or death, which ever came first

After all procedures, 280 (75.7%) anticoagulant users and 351 (69.6%) nonusers experienced at least 1 complication. The mean number of complications per patient as well as the CCI was significantly higher in anticoagulant users than in nonusers.

There was a significant difference between anticoagulant users and nonusers regarding the overall bleeding rate (29.2% vs 22%, p = 0.015). However, when stratified by severity, the rate of major bleeding defined above was similar (17.8% vs 14.1%, p = 0.129) between the two groups. Within the anticoagulant group, the lowest rate (24%) of bleeding events was in patients exposed to a DOAC. The highest rates were observed in patients exposed to a combination drug regimen (35%), followed by patients exposed to a VKA (34%) and antiplatelets (27%).

One hundred fourteen (30.8%) anticoagulant users and 111 (22%) nonusers received blood product transfusions (packed red blood cells, pooled platelets, and fresh frozen plasma) and PCCs. This difference was significant, with p = 0.003.

**Fig. 2:**
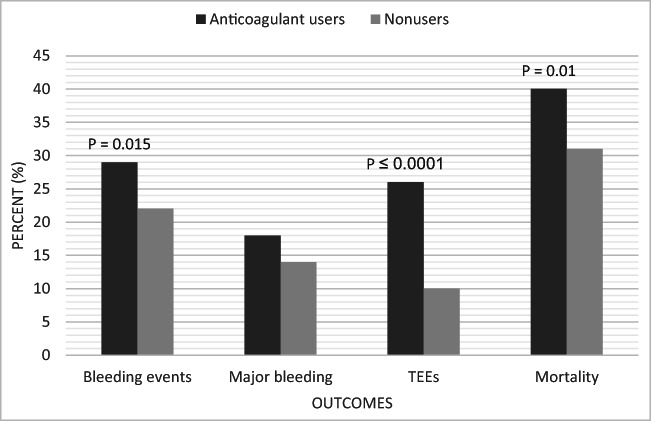
Main outcomes by group

The greatest discrepancy between groups was observed in the occurrence of TEEs, which warrants further attention. In the anticoagulant group, 25.7% had TEEs, compared with 9.7% in patients who did not use OACs (p < 0.0001) (Fig. 2)

Within the anticoagulant group, the highest rates of TEEs were observed in patients exposed to clopidogrel (50%) and a combination drug regimen (40%), and the lowest rates were observed in patients exposed to DOACs (20%) and VKAs (20%), followed by patients exposed to acetylsalicylic acid (27%). Figure (supplemental). Regarding clopidogrel, due to a very small sample size with only 10 patients included in this series, it lacks sufficient power for statistical analysis. Thus, the depicted results in supplemental figure 3 (50% TEEs and 80% mortality rate) are probably inaccurate and should therefore be interpreted with caution.

Three hundred seven (83%) anticoagulant users and 361 (71.5%) nonusers were sent to the intensive care unit (ICU). The median ICU length of stay (LOS) for anticoagulant users was 6 days (IR 1-127), which was not significantly different from the median ICU LOS of 4 days (IR 1-135) for nonusers.

Two hundred twenty-seven (61.4%) anticoagulant users and 262 (51.9%) nonusers required prolonged mechanical ventilation. The median ventilation time required was 61 h (IR 1-1713) in anticoagulant users and 59 h (IR 1-865) in nonusers.

The mortality rate in anticoagulant users was 39.7%, while that in nonusers was 31.3%. This difference was statistically significant, with p = 0.01. The overall median hospital LOS was equal between groups at 13 days.

Within the anticoagulant users cohort (AC), in-hospital mortality was 40% for those exposed to single agent antiplatelets, 37% for those exposed to a VKA, 36% for those exposed to a DOAC, and 42% for those exposed to a combination drug regimen. Overall, as depicted in Figure 3 (Supplemental), the mortality rate exceeded the incidence of bleeding and TEEs in all categories of OACs.

Multivariable logistic regression with stepwise backward selection (Tables 4, 5, and 6) was performed to determine the adjusted impact of OACs on overall bleeding events and in-hospital mortality. Other significant covariates in the model included age ≥70 years, BMI ≥ 30 kg/m^2^, hypertension, congestive heart failure, atrial fibrillation, peripheral arterial disease, diabetes, chronic renal failure, liver cirrhosis, reoperation, hemorrhage, anastomotic leaks, pneumonia, thromboembolism, and blood product transfusion.

**Table 4:** Multivariable logistic regression analysis of predictors for bleeding events

Risk factor*	OR (95% CI)	*p* value
BMI ≥ 30 kg/m^2^	1.17 (0.78–1.76)	0.444
Coronary artery disease	1.06 (0.65–1.73)	0.825
Atrial fibrillation	1.42 (0.94–2.14)	0.098
Peripheral artery disease	1.46 (0.97–2.18)	0.068
Liver cirrhosis	3.85 (2.22–6.68)	< 0.0001
Reoperation	3.17 (2.26–4.46)	< 0.0001
OAC	*1.46 (0.95–2.25)*	*0.088*

*Each risk factor represents a significant univariate predictor of bleeding events

*OR* odds ratio, *CI* confidence interval, *BMI* body mass index, *OAC* oral anticoagulation

**Table 5:** Multivariable logistic regression analysis of predictors for thromboembolic events

Risk factor*	OR (95% CI)	p value
Age ≥ 70 years	0.95 (0.60–1.51)	0.828
BMI ≥ 30 kg/m^2^	0.80 (0.49–1.30)	0.363
Hypertension	1.22 (0.72–2.06)	0.457
Congestive heart failure	1.47 (0.87–2.49)	0.153
Coronary artery disease	1.46 (0.68–1.94)	0.614
Atrial fibrillation	1.15 (0.73–1.83)	0.546
Peripheral artery disease	4.70 (3.07–7.21)	< 0.0001
Reoperation	2.20 (1.44–3.37)	< 0.001
BPT	1.96 (1.26–3.04)	0.003

*Each risk factor represents a significant univariate predictor of thromboembolism

*OR* odds ratio, *CI* confidence interval, *BMI* body mass index, *BPT* blood product transfusion

**Table 6:** Multivariable logistic regression analysis of predictors of all-cause in-hospital mortality (entire cohort)

Risk factor*	OR (95% CI)	*p* value
Age ≥70 years	1.48 (0.95–2.28)	0.081
BMI ≥30 kg/m^2^	1.59 (1.00–2.53)	0.048
Hypertension	1.11 (0.68–1.80)	0.686
Congestive heart failure	1.30 (0.78–2.17)	0.681
Atrial fibrillation	1.91 (1.21–3.03)	0.005
Peripheral artery disease	2.78 (1.74–4.44)	< 0.0001
Diabetes	1.04 (0.66–1.62)	0.872
Chronic renal failure	1.80 (1.08–3.02)	0.025
Liver cirrhosis	6.54 (3.06–13.98)	< 0.0001
Reoperation	1.50 (0.97–2.32)	0.069
Bleeding events	1.68 (0.89–3.19)	0.111
Major bleeding	1.23 (0.58–2.62)	0.585
Anastomotic leaks	1.25 (0.70–2.22)	0.448
Pneumonia	4.72 (3.12–7.15)	< 0.0001
Thromboembolism	3.68 (2.24–6.05)	< 0.0001
BPT	2.42 (1.27–4.61)	0.007
OAC	*0.45 (0.28–0.74)*	*0.002*

*Each risk factor represents a significant univariate predictor of in-hospital mortality

*OR* odds ratio, *CI* confidence interval, *BMI*, body mass index, *BPT* blood product transfusion, *OAC* oral anticoagulation

In all studied patients undergoing unplanned EL, liver cirrhosis, peripheral arterial diseases, reoperation, and blood product transfusion were independent predictors of the overall risk of bleeding or TEEs based on multivariate analysis. In this model, liver cirrhosis had the largest overall effect on mortality, followed by pneumonia, thromboembolism, peripheral arterial disease, blood product transfusion, and atrial fibrillation. Of note, as depicted in Tables 4, 5, and 6, the use of OACs was not an independent predictor of either bleeding or in-hospital mortality. The use of OACs was associated with a decreased risk of all-cause in-hospital mortality.

Overall, compared to nonusers, anticoagulant users had similar rates of major bleeding but higher rates of overall bleeding events, thromboembolism, and all-cause in-hospital mortality after EL for high-risk abdominal emergencies. In multivariate analysis, the use of OACs was not associated with an increased risk of overall bleeding events or all-cause in-hospital mortality in this subset of abdominal surgery patients.

## Discussion

Bleeding and TEEs are known complications after surgery in patients exposed to OACs. These outcomes are the central focus of many studies that included mainly elective or minor urgent surgical procedures with low associated risks. In comparison, outcome reports of EL for high-risk abdominal emergencies in patients exposed to OACs are rare in the literature. This subset of emergency surgery patients has not received sufficient attention in our view.

The present study reports the outcomes after EL for high-risk abdominal emergencies, focusing on the risks of bleeding, TEEs, and all-cause in-hospital mortality between patients who were and were not exposed to OACs.

There was a significant difference between OAC users and nonusers in the overall bleeding rate in the univariate analysis; the overall bleeding rate was higher for OAC users than nonusers. However, when stratified by severity, the rate of major bleeding as defined in the previous section was similar between the two groups. Furthermore, the rate of major bleeding was not significantly different according to the type of anticoagulant prescribed. After adjusting for other confounders, we found no association between OAC use and bleeding. Instead, in addition to reoperation, liver cirrhosis was an independent predictor of the risk of bleeding events. This may be due to an impaired synthesis of clotting factors, low platelets, and portal hypertension. These findings suggest that the increased overall bleeding rate is related to other coexisting conditions rather than to the use of OACs, and the use of OACs may not result in more bleeding in this patient population than in nonanticoagulated patients.

This finding is important because the risk of major bleeding in the setting of an emergency is perceived to depend (1) on the use of OACs and (2) on the type of anticoagulant prescribed, with the perceived highest risk in those using dual platelet inhibitors. This observation has been confirmed by other investigators in patients with AF; the addition of clopidogrel to acetylsalicylic acid reduced the risk of stroke but increased the risk of fatal bleeding in patients with AF 16.

In previous studies, the incidence of bleeding events exceeded the mortality rate in those undergoing elective surgical procedures. However, the opposite was observed after emergency surgery, and death was more common than bleeding 7. This is in agreement with our data that showed a significantly higher in-hospital mortality rate than bleeding rate in both groups after EL for high-risk emergencies.

In the current study, thromboembolism increased the odds of mortality by more than threefold. Indeed, our data suggest that postoperative thromboembolism warrants a higher degree of concern than bleeding and should be considered a potentially lethal event in patients undergoing surgery for high-risk abdominal emergencies. Of course, we are not understating how severe and life-threatening bleeding can be in patients, but as indicated by others 17, bleeding is generally easier to treat than TEEs.

The high rate of TEEs in chronic OAC users in this study suggests that despite bridging therapy (40% were bridged), the temporary interruption of OACs combined with a transfusion of blood products (BPT) may be a precipitating event. For example, the rates of TEEs and in-hospital mortality in patients who required blood product transfusion were 42% and 66%, respectively. By multivariate analysis, BPT was associated with a 2-fold increase in the odds of TEEs and a 2.4-fold increase in the rate of all-cause in-hospital mortality, indicating that BPT is a significant risk factor for a poor outcome after surgery. This is in agreement with other studies that found bridging anticoagulation therapy during an interruption of OACs and BPT were associated with a higher risk of TEEs and other adverse events 5, 17–19.

All-cause in-hospital mortality was significantly higher in patients exposed to OACs than in nonexposed patients, but in multivariate analysis, anticoagulant exposure was not associated with an increased risk of in-hospital mortality. Indeed, it was associated with a decreased risk of mortality. Therefore, given the comparable rate of major bleeding, preoperative OAC exposure may not explain the difference in mortality rate. In addition, the mortality rate in our study was notably high for this highly selected patient cohort. However, if we consider our entire primary emergency cohort including those patients with minor emergencies, the overall mortality rate is 14.5%, which is within the range of mortality rates reported in the literature 2, 7–9. Therefore, the high mortality rate indicated in this study is relative and attributable to the risk-based approach of patient selection. We included only consecutive multimorbid patients with high-risk emergencies. These patients are at high risk of procedural adverse events and tend to have septic complications with multiple organ dysfunctions that inevitably lead to death 12, 13. Sepsis with multiple organ failure was documented in 84.2% (256 of 304 deaths) of the fatality cases.

Overall, based on our results, the continued use of OACs is more protective than harmful regarding the overall outcomes in this subset of patients. The reduction in major bleeding events caused by the interruption of OACs and transfusion of reversal agents was offset by an increase in fatal TEEs.

Our study has certain limitations worth discussing. First, the data were collected in a retrospective fashion and are susceptible to errors in recording. Second, because we relied partly on information from patients who required unplanned emergency surgery, we were not able to ascertain patient compliance and adherence to their medication, as well as the exact stop date before surgery. However, we anticipate that this affects only a small proportion of patients because the past medical history of the majority of studied patients was recorded at our institution. Third, we could not assess the use of reversal agents to DOACs for anticoagulant reversal, as the use of such agents was not consistently documented in our database. Fourth, the disproportionate distribution and low number of patients in each anticoagulant category may limit the power of our analysis to detect small differences. Fifth, along these lines, the presence of comorbid conditions was significantly higher in anticoagulant users than in nonusers. However, in multivariate analysis, we adjusted for all of these variables to predict outcomes based on adjusted variables. Therefore, once a multivariate analysis is completed, it would have adjusted for the difference.

Despite these limitations, we feel that this study is of clinical importance because it addresses the important issue of chronic anticoagulant exposure and provides pertinent data on the outcomes of a selected cohort of patients who underwent surgery for high-risk abdominal emergencies. The presence of a control group for comparison to define the magnitude of negative outcomes of emergency surgery with and without exposure to chronic OACs is also a strength of this study.

## Conclusions

Among patients who underwent surgery for high-risk abdominal emergencies, patients exposed to OACs and antiplatelet agents had similar rates of major bleeding but higher rates of overall bleeding events, TEEs, and all-cause in-hospital mortality than nonanticoagulated patients. Multivariable logistic regression analysis showed that the use of OACs was not associated with a significantly increased risk of overall bleeding events. Indeed, our data suggest that it was associated with a decreased in-hospital mortality rate, indicating a protective effect. Based on these data, the unfavorable outcomes after surgery for high-risk abdominal emergencies indicated in the current study do not appear to be mediated by the use of OACs.

## Supplementary Information

ESM 1 (PDF 96 kb)

ESM 2 (PDF 65 kb)
