# Supplementary figures and images for: The Effects of Oral Anticoagulant Exposure on the Surgical Outcomes of Patients Undergoing Surgery for High-Risk Abdominal Emergencies
Source: J Gastrointest Surg. 2021 Mar 22;25(11):2939–47. doi: 10.1007/s11605-021-04964-9 (PMC8602169; doi:10.1007/s11605-021-04964-9)

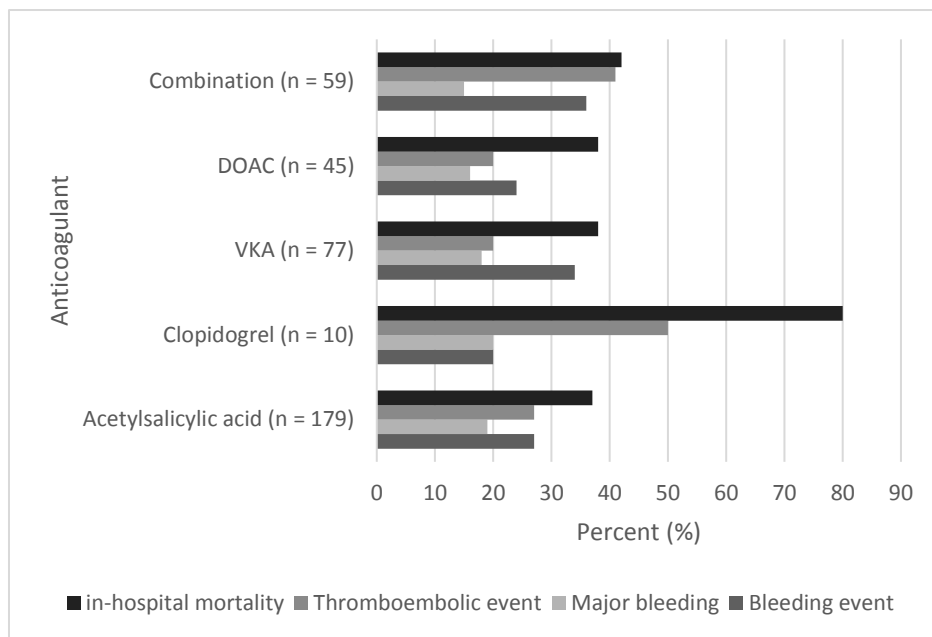

**Figure (Supplemental).** Main outcomes by anticoagulant category

Supplement: Supplementary file 1 — (PDF 96 kb) [file 11605_2021_4964_MOESM1_ESM.pdf]
