# Supplementary material for: The Effects of Oral Anticoagulant Exposure on the Surgical Outcomes of Patients Undergoing Surgery for High-Risk Abdominal Emergencies
Source: J Gastrointest Surg. 2021 Mar 22;25(11):2939–47. doi: 10.1007/s11605-021-04964-9 (PMC8602169; doi:10.1007/s11605-021-04964-9)
Supplement: Supplementary file 2 — (PDF 65 kb) [file 11605_2021_4964_MOESM2_ESM.pdf]

Table (Supplemental). Overall outcomes by type of surgery

	Entire cohort, N = 875			
Index Procedure	n (%)	Bleeding events	TEEs	Mortality rate
Closure of viscus organ	130 (14.9)	16 (12.3)	8 (6.2)	37 (28.5)
Right colectomy including subtotal resection	112 (12.8)	39 (34.8)	33 (29.5)	55 (49.1)
Multivisceral procedures	112 (12.8)	37 (33.0)	24 (21.4)	43 (38.4)
Small bowel resection	107 (12.2)	32 (29.9)	25 (23.4)	35 (32.7)
Laparotomy with extensive adhesiolysis	104 (11.9)	11 (10.6)	8 (7.7)	22 (21.2)
Hartmann’s procedure	88 (10.1)	22 (25.0)	9 (10.2)	32 (36.4)
Surgery for complicated cholecystitis	50 (5.7)	6 (12.0)	2 (4.0)	6 (12.0)
Hemostasis	45 (5.1)	29 (64.4)	7 (15.6)	23 (51.1)
Surgery for complicated appendicitis	29 (3.3)	1 (3.4)	0 (0.0)	1 (3.4)
Laparotomy only	27 (3.1)	6 (22.2)	11 (40.7)	25 (92.6)
Repair of intestinal perforation with formation of stoma	15 (1.7)	(20.0)	2 (13.3)	6 (40.0)
Vascular repair via laparotomy and bowel resection	14 (1.6)	7 (50.0)	11 (78.6)	7 (50.0)
Hernia repair	13 (1.5)	1 (7.7)	0 (0.0)	3 (23.1)
Left colectomy including anterior resection	12 (1.4)	2 (16.7)	1 (8.3)	3 (20.0)
Oesophagogastric resection of any type	11 (1.3)	7 (63.6)	2 (18.2)	6 (42.9)
Colectomy unspecified	3 (0.3)	0 (0.0)	1 (33.3)	0 (0.0)
Splenectomy	3 (0.3)	0 (0.0)	0 (0.0)	0 (0.0)

Numbers in bracket show values presented in n (%) unless noted otherwise.
